# Klatskin-Like Lesions

**DOI:** 10.1155/2012/107519

**Published:** 2012-06-28

**Authors:** M. P. Senthil Kumar, R. Marudanayagam

**Affiliations:** ^1^The Liver Unit, Queen Elizabeth Hospital Birmingham, Edgbaston, Birmingham B15 2TH, UK; ^2^Department of HPB Surgery and Liver Transplantation, Queen Elizabeth Hospital Birmingham, 3rd Floor Nuffield House, Edgbaston, Birmingham B15 2TH, UK

## Abstract

Hilar cholangiocarcinoma, also known as Klatskin tumour, is the commonest type of cholangiocarcinoma. It poses unique problems in the diagnosis and management because of its anatomical location. Curative surgery in the form of major hepatic resection entails significant morbidity. About 5–15% of specimens resected for presumed Klatskin tumour prove not to be cholangiocarcinomas. There are a number of inflammatory, infective, vascular, and other pathologies, which have overlapping clinical and radiological features with a Klatskin tumour, leading to misinterpretation. This paper aims to summarise the features of such Klatskin-like lesions that have been reported in surgical literature.

## 1. Introduction

Hilar cholangiocarcinoma, also known as Altemeier-Klatskin tumour is a primary malignancy of the liver occurring at the confluence of the bile ducts, first reported by Altemeier et al. in 1957 and characterised by Klatskin in 1965 [1, 2]. Occurring within 2 cm of the hilar confluence, it accounts for about 50–70% of all cholangiocarcinomas [3]. 

Resection and in selected patients, transplantation offers the best chance of cure in Klatskin tumours. Hence, early diagnosis is vital for a radical surgical approach to be feasible and effective. Equally, it is ideal, though not always possible, to have an established diagnosis of cancer or a strong probability of malignancy before embarking on a radical liver resection in view of the potential morbidity and mortality. Klatskin tumours have to be differentiated from a number of benign pathologies and some malignant lesions that mimic the clinical presentation and the radiological appearances (Table 1). These have been variously called “Klatskin-mimicking lesions,” “the malignant masquerade,” and “pseudo-Klatskin tumours” [4, 5]. In most large series of hilar strictures, operated on, with a preoperative diagnosis of cholangiocarcinoma (CCA), the rate of benign lesions on final histopathology ranges from 5 to 15%, reaching up to a third in some reports (Table 1) [6–10]. 

Hilar cholangiocarcinoma has three morphological types: periductal infiltrative, polypoid, and exophytic (mass forming), depending on the predominant pattern of spread in relation to the duct wall. Infiltrating CCA is the commonest type of hilar CCA (70%) and typically appears as a focal thickening of bile duct with hyperattenuation on imaging. Polypoid CCA appears as an intraluminal hypoattenuating lesion. Exophytic hilar CCA is typically seen as a hypodense mass lesion with rim enhancement. Tumour markers such as CA19-9, IL-6, and neutrophil gelatinase-associated lipocalin (NGAL), although are typically raised in CCA, do not have sufficient discriminatory power to be clinically useful in Klatskin mimicking lesions [8–11]. ERCP and PTC are also useful to clarify the anatomy. Brush cytology, though it has a high specificity, suffers from a low sensitivity. Cholangioscopy with cytology and confocal laser endomicroscopy are promising evolving technologies in the evaluation of indeterminate biliary strictures [12, 13].

Multimodal imaging to include multidetector CT, MRI, and MRCP is the key. In general, vascular involvement, involvement of secondary biliary radicles, duct wall thickness >4 mm, lobar atrophy are all pointers to a cholangiocarcinoma but are not diagnostic [14, 15]. There are a few confounding issues which need attention while interpreting the clinical and imaging features in Klatskin tumours. Some of the Klatskin mimicking lesions such as tuberculosis, sarcoidosis, lymphoma, metastasis may have prominent hilar lymphadenopathy leading to a misdiagnosis of an advanced cholangiocarcinoma. Some diseases such as primary sclerosing cholangitis, intrahepatic stones, and oriental cholangiohepatitis which by themselves may mimic a Klatskin lesion also are high risk factors for the development of cholangiocarcinoma and may be harbouring them at presentation.

Hilar lesions should be interpreted in the clinical context that they present. In addition to a detailed history of current symptoms past medical and surgical history is important. Biochemical, hematological, serological, and radiological evidence of involvement of other organ systems should be sought, especially in young patients, as there may be specific clues in certain disorders such as sarcoidosis, connective tissue disorders, IgG4-related disease, eosinophilic cholangiopathy, HIV, and tuberculosis. It is important to recognize them as there are alternative treatments available. In some disorders such as portal biliopathy surgery may be dangerous and may be avoided with the correct preoperative diagnosis. 

The aim of this paper is to highlight the presentation and features of various Klatskin-like lesions that have been and may be interpreted as hilar CCA. A systematic medline search was performed for relevant publications between 1966 and 2012, using the terms hilar cholangiocarcinoma, Klatskin tumour, Klatskin-mimicking lesions, and pseudo-Klatskin tumour. Retrieved publications and their references were then scrutinized for reports of lesions that mimicked Klatskin tumours. A summary of the key lesions is presented (Table 2).

## 2. Dominant Stricture of Primary Sclerosing Cholangitis (PSC)

PSC is a chronic cholestatic disorder of possible autoimmune etiology in which a localized high grade stricture in the intra- or extrahepatic bile ducts may be the presenting feature in up to 20% of patients [16]. Though PSC classically manifests as multifocal strictures and dilatations, which are often discontinuous, leading to a beaded appearance on imaging, a dominant stricture at the hilum may be mistaken for a CCA. Central to the interpretation of strictures in PSC is the fact that the lifetime risk of cholangiocarcinoma in PSC is up to 25%. Nearly two-thirds of patients who develop a cholangiocarcinoma in the context of PSC have a dominant stricture, and hilar lesions in PSC have a higher risk of being malignant [16, 17]. 

Clinically, the association of inflammatory bowel disease (in up to 80% of patients) is a useful clue. Serum tumour markers and noninvasive imaging modalities have a low sensitivity and specificity in differentiating benign from malignant stricture. Bile CEA levels >30 ng/ml may be a useful discriminator [18]. While conventional brush cytology at ERCP or PTC suffers from a high false negative rate, transpapillary cholangioscopy with brush cytology is a sensitive test and also retains a high specificity [19, 20]. 

## 3. Intrahepatic Stones and Recurrent Pyogenic Cholangitis

Though hepatolithiasis may complicate any longstanding stricture or ductal dilatation, it is a classical feature of the syndrome of recurrent pyogenic cholangitis (RPC). Endemic in Southeast Asia and first described by Digby in 1930, RPC is a syndrome of recurrent infections of the biliary tree associated with intrahepatic strictures and hepatolithiasis [21]. It presents between the 3rd and 5th decades with no gender preponderance, with stones typically occurring in the left lateral segment and the right posterior segments. Classical features are Charcot's triad (recurrent fever, right upper quadrant pain and jaundice) and Reynold's pentad (+ hypotension and altered sensorium). 

The etiology of intrahepatic stones is complex with an interplay of metabolic abnormalities, poor bile flow and stasis, infections or infestations, ductal mucin secretion, proliferative cholangitis, all playing a role. Ascaris and liver flukes such as Clonorchis, Opisthorchis, and Fasciola are thought to initiate the biliary injury, which is propagated by the stones, inflammatory strictures, and the invariable occurrence of multiple cycles of cholangitis. However, an alternative view is that E coli infection coupled with a low protein diet favours deconjugation of bilirubin in the bile ducts leading to sludge and later stones [22]. 

There are two common types of intrahepatic stones—brown pigment and cholesterol which elicit three types of inflammation—suppurative cholangitis, chronic proliferative cholangitis and chronic granulomatous cholangitis. Pigment stones with calcium bilirubinate as the chief component are the commonest type of stones, and about 90% of these are hyperintense on plain CT. The other classical features of RPC on imaging are central duct dilatation with rapid tapering (arrow head sign), poor arborisation of bile ducts in the periphery, and segmental nonfilling of ducts (absent duct sign) [22]. Hilar stricture due to hepatolithiasis has been misinterpreted as cholangiocarcinoma [23, 24]. Stones may be imaging occult especially with contrast CT, as they may be isodense. Noncontrast CT is more sensitive. MRI is the best modality to delineate the stones, the extent of the stricture and lobar atrophy. 

## 4. Mirizzi Syndrome

Named after Pablo Luis Mirizzi, who described it in 1948, it is the obstruction of the common hepatic or bile duct by a gallstone impacted in the cystic duct or Hartmann's pouch of the gall bladder. Inflammation around the hilum may result in a stricture. Presence of a large impacted stone is an important pointer to the diagnosis, but gallstones are not always seen on a CT, and the imaging findings may mimic a periductal infiltrating type of cholangiocarcinoma [14]. MR cholangiography is the investigation of choice as it often identifies the gallstone, the extrinsic compression, the dilated proximal ducts and normal distal ducts which are the defining features of Mirizzi syndrome. 

## 5. Inflammatory-Infiltrative

### 5.1. Inflammatory Pseudotumour (IPT)

First recognized in 1939 in the lungs, hepatic IPT was first described by Pack and Baker in 1953 [25]. IPT is a relatively rare cause of a hilar mass lesion, which closely mimics a cholangiocarcinoma, with only about 200 reports in literature. IPT occurs in young adults and children and is commoner in the lungs, stomach, omentum, and mesentery among other sites. It is a benign nonneoplastic proliferative lesion characterized histologically by a heterogenous inflammatory infiltrate of plasma cells, lymphocytes, macrophages, eosinophils, and dendritic cells amidst a myofibroblastic background [26]. Macroscopically it forms a nonencapsulated mass lesion in the liver, which ranges from grey-white to brown-tan in appearance. Though an inflammatory response to efflux of toxic bile acids is thought to contribute to the pathogenesis, the exact etiology is unknown [27]. There are associations with Crohn's disease, phlebitis, Epstein-Barr virus infection, autoimmune pancreatitis, PSC, and recurrent pyogenic cholangitis [27, 28]. Rare instances of multiple IPTs and invasion of hepatic veins have been reported in literature [29]. 

On CT and MRI, there is typically no arterial phase enhancement, but there is delayed peripheral enhancement in the portal venous phase [30, 31]. Imaging modalities including CT scan, liver-specific MRI, and PET scan are not reliable in differentiating IPT from hilar cholangiocarcinoma [28, 31]. CEA and AFP are usually normal in IPT, while CA19-9 may be mildly elevated in some patients. The prognosis is good, and spontaneous resolution has been reported. Corticosteroids, antibiotics, and nonsteroidal anti-inflammatory drugs have been used with inconsistent effect. 

### 5.2. IgG4-Related Sclerosing Cholangiopathy (ISC)

IgG4-related autoimmune disease is an inflammatory multisystem disorder of which cholangiopathy may be one of the manifestations. The tissues affected range from pancreas (autoimmune pancreatitis), liver and bile ducts (autoimmune hepatitis; cholangiopathy), salivary glands (chronic sclerosing sialadenitis), lacrimal glands (Mikulicz's disease) retroperitoneum (retroperitoneal fibrosis), mediastinum (sclerosing mediastinitis) and kidneys [32]. It is characterized by infiltration of tissues by IgG4-positive plasma cells and associated with elevated levels of serum IgG4. 

ISC is of two distinct types: a diffuse sclerosing cholangitis pattern and a hilar pseudotumour pattern [32]. Typically it is a disease of the large bile ducts. The distinguishing features of ISC are concentric bile duct thickening, smooth strictures, multifocal involvement, minimal proximal dilatation and the absence of ectasia, diverticula and pruning [33].

More than 90% of the patients with ISC have autoimmune pancreatitis, and it has been called IgG4-related sclerosing pancreatocholangitis [34–36]. The pancreatitis often precedes the presentation of cholangitis although it may follow it. An Ig-G4 level of >140 mg/l is considered significant for the diagnosis of Ig-G4 related disease, while a level >300 mg/l confers high specificity [32]. Other serological markers include hypergammaglobulinemia, antinuclear antibody and peripheral eosinophilia [32]. 

Imaging modalities including CT, MRI, and PET scans are not useful in differentiating a hilar cholangiocarcinoma from Ig-G4 cholangiopathy with certainty [32]. However, they may provide indirect evidence by detecting the associated autoimmune pancreatitis, which has distinct and diagnostic imaging features. Brush cytology is an unreliable discriminator due to the frequency of false negative and false positive results [37]. Histology, however, is reliable. The histological hallmarks of IgG4- related disease are lymphoplasmacytic infiltration especially IgG4-positive plasma cells, storiform fibrosis, obliterative phlebitis, and eosinophilia. In patients presenting with biliary strictures options to achieve histological diagnosis include a percutaneous or endoscopic biopsy of the stricture or mass lesion or a biopsy of the ampulla of vater [32]. 

Ig-G4 cholangiopathy runs a benign course, and the associated pancreatitis may resolve spontaneously. Ig-G4 cholangiopathy responds well to oral corticosteroids. The recommended dose based on extrapolation from the studies on autoimmune pancreatitis is prednisolone 0.6 mg/kg/d which may be tapered over 3–6 months [35, 36, 38]. Rituximab is an option for steroid refractory disease [39]. 

### 5.3. Eosinophilic Cholangiopathy (EC)

Eosinophilic cholangiopathy or cholangitis is characterized by a dense transmural inflammatory infiltration of the biliary tract by eosinophils, leading to strictures and obstructive jaundice. Mass lesions are uncommon.

Although Albot et al. described eosinophilic cholecystitis in 1949, Butler et al. in 1985 were the first to characterize eosinophilic cholangiopathy [40, 41]. EC is rare with only about 10 cases reported in literature. Though EC may occur as an isolated phenomenon, it often manifests as a part of a systemic syndrome involving other organs and tissues (bone marrow, kidney, ureters, stomach, bowel pancreas, and lymph nodes). In the biliary tree, gall bladder involvement (eosinophilic cholecystitis) is slightly more common than ductal involvement. 

Peripheral eosinophilia is an important clue to the possibility but is not universal feature. In patients with eosinophilic gastroenteritis and biliary stricture, the presentation may mimic primary sclerosing cholangitis with ulcerative colitis [42]. Patients with eosinophilic gastroenteritis who have serosal involvement may develop ascites, which may clinically mimic advanced cholangiocarcinoma. Ascitic fluid cytology is eosinophil rich and is a useful diagnostic test [42]. 

Pathologically, the differential diagnosis in view of the portal eosinophilic infiltrates includes parasitic, fungal and drug-induced liver disease, as well as primary sclerosing cholangitis, allograft rejection, autoimmune cholangitis, and primary biliary cirrhosis, depending on the context. 

The etiology and pathogenesis of EC are poorly understood. There are no specific or characteristic radiological or serological tests. However, the prognosis is good with a quick and, often, a sustained response to steroids. For example, Vauthey et al. report a patient who received prednisolone at the dose of 40 mg/day for 8 weeks and had a complete resolution of the biliary lesion and was well at 18-months followup [43]. 

### 5.4. Follicular Cholangitis (FC)

FC is another lymphoplasmacytic infiltrative process of unknown etiology, which is similar to ISC but in contrast to which, the characteristic feature is the presence of periductal lymphoid follicles. It was first described by Aoki et al., in 2003, and there have been a few case reports since, mostly in the eastern literature [44–47]. Patients are usually more than 40 years of age, and there is no gender preponderance. The hilar bile ducts are typically involved. It may affect the pancreas concurrently (follicular pancreatocholangitis). Plasma IgG4 levels are not raised nor is there a prominence of IgG4-positive plasma cells on histology. There are no disease associations, no specific serum markers, or diagnostic imaging features. Patients usually undergo resection for suspected cancer. There are no reported recurrences. The natural history and the response to steroids are unknown. 

### 5.5. Mast Cell Cholangiopathy

Mast cells are fibrogenic and contribute to the pathogenesis of certain hepatic diseases where fibrosis plays a key role such as alcoholic liver disease and PSC. Though diffuse mast cell infiltration of the liver in systemic mastocytosis resulting in intrahepatic cholestasis is not uncommon, it is an extremely rare cause of secondary sclerosing cholangitis manifesting as ductal lesions, with only 2 case reports in literature [48, 49]. The patients were both women over 70 years, had systemic mastocytosis, and presented with jaundice. One patient had ascites while the other had lytic bone lesions. Bile duct thickening on CT and multiple ductal lesions on cholangiography were present. Urinary histamine and tryptase levels are raised in mastocytosis and are useful in diagnosis, as is a bone marrow biopsy. 

### 5.6. Xanthogranulomatous Cholangitis (XGC)

Xanthogranulomatous cholecystitis is an uncommon, but well-characterized chronic invasive inflammatory process of the gall bladder which may mimic gall bladder carcinoma. In a particularly aggressive form of xanthogranulomatous cholecystitis, the inflammatory fibrosis spreads to contiguous tissues and organs [50]. When this involves the hilar bile ducts, it may simulate a Klatskin tumour. Xanthogranulomatous cholecystitis almost always occurs in the presence of gall stones and is associated with a significant thickening of gall bladder wall, which is unusual in cholangiocarcinoma. 

In XGC, an even rarer condition, a similar inflammation typified by infiltration of the bile duct wall by foamy macrophages with inflammatory infiltrate and fibrosis occurs, leading to thickening and smooth strictures involving the large bile ducts. XGC may occur alone or in association with xanthogranulomatous cholecystitis and may occur in children as well as adults [51–54]. There is no gender predilection. Hilar lesions are clinically indistinguishable to Klatskin tumours and have been diagnosed with certainty only after hepatic resection [54]. 

### 5.7. Sarcoidosis

Sarcoidosis is a steroid responsive multisystem granulomatous disorder of uncertain etiology which is characterized by noncaseating epitheloid granulomas, commonly affecting the lungs, lymph nodes, eyes, and the skin. Hepatic manifestations were first described by Klatskin and Yesner in 1950 and include granulomatous hepatitis, cholestasis, cirrhosis, portal fibrosis leading to presinusoidal portal hypertension, Budd-Chiari syndrome, adult ductopenia-like syndrome, and rarely chronic granulomatous sclerosing cholangitis with ductal strictures [55–57]. Nodal involvement with hilar stricture may closely resemble a hilar cholangiocarcinoma [57]. Moreover sarcoid-like nodal changes may be seen in other malignancies further confounding the issue [58]. Hepatic sarcoidosis detectable on liver biopsies may also be associated with PSC and PBC [59]. 

Sarcoidosis commonly occurs in women in their 3rd or 4th decades, and hepatic sarcoidosis almost always has concurrent pulmonary involvement. PET CT has been shown to be useful in targeting nodes for biopsy. Spyglass cholangioscopy with directed biopsy may clinch the diagnosis with a high degree of accuracy [57]. Hypercalcemia and elevated serum angiotensin-converting enzyme are useful corroborative tests but are neither sensitive nor specific in hepatic sarcoidosis. The mainstay of treatment of symptomatic strictures in sarcoidosis is drainage. Ursodeoxycholic acid and corticosteroids (prednisolone 40–60 mg/day for 6 months) may benefit cholestasis but may not resolve associated fibrosis or ductopenia [57, 59].

## 6. Infective

### 6.1. Cholangiopathy of  Immunosuppression

HIV infection, especially when it has progressed to full-blown AIDS, is associated with characteristic biliary abnormalities, which collectively are known as AIDS cholangiopathy, first recognized in 1986 [60]. It is more common in advanced disease, poorly treated disease, and in patients with low CD4 counts (<135/mm3) and hence is a marker of poor prognosis [61, 62]. Sclerosing cholangitis is seen in more than 70% of patients with AIDS cholangiopathy, most commonly involving the large intrahepatic ducts, either alone or in association with papillary stenosis or extrahepatic duct strictures [62, 63]. There is often an elevated alkaline phosphatase, and most patients are symptomatic. Treatment is by papillotomy where necessary, and imaging directed dilatation and stenting of strictures. Specific therapy targeted against a host of opportunistic pathogens implicated such as cytomegalovirus, cryptosporidium, mycobacteria does not seem to affect the long-term outcome [64].

Sclerosing cholangitis is the commonest hepatobiliary complication in children with primary immunodeficiency. Cholangiographic abnormalities were found in 60% of patients with clinical evidence of liver disease. Cryptosporidium parvum infection was the major pathogen implicated. These patients are also prone for biliary malignancies. Hematopoietic stem cell transplant in the early stages and combined liver transplant in late stages have a therapeutic role [65, 66]. 

### 6.2. Bacterial Infections

Rarely multiple pyogenic liver abscesses may cause ductal abnormalities [67]. This has also been reported in relation to systemic gram-positive sepsis and infection with *E. coli* [68, 69]. Although the clinical context makes the diagnosis self-evident, if the underlying primary cause such as diverticulitis is clinically subtle, and the presentation is with jaundice or deranged liver function tests, then ductal dilatation and strictures on imaging may be misconstrued as possible cholangiocarcinoma. 

### 6.3. Tuberculosis

Four types of hepatobiliary tuberculosis caused by Mycobacterium tuberculosis are recognized—military tuberculosis of the liver, granulomatous hepatitis, local parenchymal disease, and the rarer focal sclerosing type due to direct involvement of bile ducts resulting in strictures [70, 71]. In addition periportal tuberculous lymphadenitis is a well-documented cause of hilar biliary obstruction, which may mimic a cholangiocarcinoma [72]. 

Though hepatic involvement in TB is common as a part of disseminated disease, isolated biliary TB affecting the ducts is rare. Hilar strictures in biliary TB are difficult to distinguish from cholangiocarcinoma, but the younger age (<40 years in two-thirds), history of low-grade fever before the onset of jaundice, presence of extra hepatic TB and calcifications in the liver are suggestive [72]. Brush cytology demonstrating acid-fast bacilli (AFB), biopsies showing caseating granulomas, and bile PCR for AFB are useful tests [71, 73, 74]. Hilar nodal disease and compression from granulomas may also cause ductal abnormalities on imaging. Fibrotic strictures may need specific intervention in addition to the standard antituberculous chemotherapy. 

There has been a case report of systemic Mycobacterium genavense causing sclerosing cholangitis [75]. 

### 6.4. Fungal

Invasive fungal infections involving the liver are often seen in immunocompromised patients and often are a marker of a disseminated infection. The microabscesses of candidiasis and the granulomas of histoplasmosis, aspergillosis, blastomycosis are well studied, but, in general, these do not mimic a cholangiocarcinoma. 

However, in mucormycosis insidious progression of focal liver lesions may present with mass lesions and hilar ductal strictures [76]. There have also been a few case reports of disseminated cryptococcosis manifesting as cholangitis [77–83]. This may even occur in the immunocompetent host. Some patients have ductal lesions with or without lymphadenopathy mimicking hilar cholangiocarcinoma [83]. Bile culture, nodal FNA, and culture, serum cryptococcal antigen screen are useful diagnostic tools. The lesions respond to antifungal treatment. 

### 6.5. Parasitic

Parasites such as ascaris and liver flukes are known to be associated with the syndrome of oriental cholangiohepatitis. Rarely, focal duct strictures of the major hepatic ducts resembling a cholangiocarcinoma may be caused by Clonorchis infestation [84]. 

## 7. Vascular

### 7.1. Portal Hypertensive Biliopathy (PHB)

There are recognizable changes that occur in the biliary tree as a consequence of portal hypertension and cavernomatous transformation of the portal vein, which have been collectively and variously labeled as portal hypertensive biliopathy, cholangiopathy associated with portal hypertension and portal cavernoma-associated cholangiopathy [85]. 

PHB in essence is due to the pressure and ischemia on the biliary tree from the engorged collateral veins that evolve in portal hypertension. The epicholedochal venous plexus of Saint (a fine reticular web that hugs the bile ducts) and the paracholedochal venous plexus of Petren (a longitudinally oriented network of larger caliber veins) are central to the pathophysiology. The epicholedochal plexus is thought to cause fine irregularities in the bile ducts, while the paracholedochal plexus causes the mass effect. With chronicity, a solid connective tissue scaffold forms around the leash of collaterals encasing the bile duct [85–87]. 

PHB is more common in extrahepatic portal venous obstruction, occurring in more than 80% of patients than in cirrhosis, where the frequency is up to 30%. There is a male predominance and being a slowly progressive disease, they often present in the fourth decade of life. Smooth strictures and segmental dilatations are typical and may be intrahepatic or extrahepatic. Other abnormalities include angulation, indentation, pruning, and clustering of intrahepatic ducts [85]. In cirrhotics the strictures are predominantly intrahepatic, while in extrahepatic portal venous obstruction, they are both extra- and intrahepatic [88]. The left duct is involved nearly twice as commonly as the right duct [85]. 

A stricture at the hilum with the mass effect caused by the cavernoma mimics a cholangiocarcinoma and has been termed “pseudocholangiocarcinoma sign” [89]. Intrahepatic strictures in PHB may resemble primary sclerosing cholangitis at cholangiography, and the term “pseudosclerosing cholangitis” has been used to describe this [90]. The key distinguishing characteristic of the strictures in PBH is that they are smooth as opposed to the typically irregular contour seen in cholangiocarcinoma. 

There is an increased incidence of biliary lithiasis (about 20%), and this in fact may bring the problem to light. Most patients, however, are asymptomatic and are often discovered incidentally on imaging for other clinical problems. About a fifth to a third of patients become symptomatic with pain, jaundice, recurrent cholangitis or present with significantly deranged liver function tests [85]. Though ultrasound, either transabdominal or endoscopic, is useful to assess the varices, MR portography and MR cholangiography are the investigations of choice in suspected PHB. 

Portosystemic shunt is known to cause resolution of the varices and the biliary strictures, and hence this should be considered as an important therapeutic option [87, 91–93]. Symptomatic patients will require percutaneous or endoscopic biliary dilatation and repeated stent changes every 4–6 months. 

A special challenge in these patients is the risk of hemobilia after biliary interventions. Periampullary varices increase the risk of postsphincterotomy bleed, and intracholedochal varices may mimic stones on a cholangiogram and may result in significant bleeding if traumatised by a Dormia basket. Open surgery may equally be difficult due to a wall of varices around the bile duct and at the hilum making safe access a challenge. For distal lesions, it has been suggested that these patients undergo a portosystemic shunt (mesocaval; lienorenal; TIPPS), if there is a shuntable vein, as the first stage and then considered for a second stage biliary enteric anastomosis after stone clearance [85]. Selected patients may benefit from liver transplantation [94]. 

### 7.2. Ischaemic Cholangiopathy

Ischemic injury results in tissue death resulting ultimately in fibrosis. The biliary tree is solely dependent on the hepatic arterial flow unlike the parenchyma and hence is very susceptible to ischaemic insults. The commonest region to be affected by ischemic cholangiopathy is the midcommon bile duct followed by the hepatic ductal confluence [95]. 

Multifocal nonanastomotic ischaemic biliary stricture following liver transplant is the prototype ischemic cholangiopathy. This is most evident in donation after cardiac death (DCD) grafts with long warm or cold ischaemic time and after hepatic artery thrombosis. But because of the context there is hardly any uncertainty about the diagnosis. 

Similarly strictures following iatrogenic bile duct injury at cholecystectomy are evident from the clinical context. Inadvertent right hepatic arterial injury at laparoscopic cholecystectomy is estimated to occur in 7% of cholecystectomies and in about 25% of patients who have a bile duct injury [96]. The contribution of ischemia to strictures following bile duct reconstruction after repair of iatrogenic bile duct injuries is difficult to estimate. However, it is known that a certain proportion of patients develop hilar and intrahepatic strictures and consequent recurrent cholangitis ultimately needing liver resection [97]. 

A secondary sclerosing cholangitis of the critically ill, of presumed ischaemic origin has been recognized [98, 99]. This typically occurs in patients who have had septic shock of a diverse etiology and consequently had to spend a prolonged time in the intensive care unit with respiratory and cardiovascular support. Enterococcus faecium was consistently isolated from many patients in one study [99]. 

Ischemic biliary strictures have been described in hepatic artery atherosclerosis [100]; sickle cell disease [101], polyarteritis nodosa [102]; paroxysmal nocturnal hemoglobinuria [103]; hereditary haemorrhagic telangiectasia [104]; henoch-schonlein purpura [105]; systemic lupus erythematosus and scleroderma [95]. On occasion, when they occur close to the hilum, they may potentially raise the suspicion of a malignant stricture. External beam radiotherapy-induced strictures have been described in the extrahepatic bile duct, and intrahepatic strictures may occur after selective internal radiation therapy (SIRT) or after transcatheter hepatic arterial embolization, but often the clinical context makes the diagnosis apparent [106–108].

## 8. Trauma

Blunt trauma is a rare cause of biliary strictures, and the commonest site is the supraduodenal CBD [109, 110]. However, hilar stricture resembling a Klatskin tumour has been reported [111]. Presentation often is delayed for many weeks to years. Interestingly, remote severe trauma or severe sepsis, not directly involving the biliary tree, may also result in a posttraumatic sclerosing cholangitis [98, 112]. The pathogenesis is presumed to have an ischemic origin as most patients at least in one series had hemodynamic instability following trauma [113]. 

## 9. Toxic

Chemotherapy-induced biliary sclerosis (CIBS) is well documented after intra-arterial infusional chemotherapy with 5-FU and may occur in up to half the patients receiving the treatment [114]. The hilar bifurcation and the proximal common hepatic duct are the commonest sites [115, 116]. Intrahepatic infiltration of cisplatin, mitomycin C, and formaldehyde are also known to lead to biliary sclerosing lesions [28]. Direct toxicity and vasculitis are thought to be the mechanisms. 

## 10. Tumours

A number of malignancies involving the hilum may mimic a CCA. The key among them is gall bladder carcinoma, hepatocellular carcinoma, neuroendocrine tumours, colorectal metastasis, lymphoma, leukemia, and myeloma. 

Gall bladder cancers usually have a dominant lesion in the gall bladder, but a primary arising from region of the neck and infiltrating the hilum is difficult to differentiate from a Klatskin tumour. Infiltrating HCC typically has early arterial phase enhancement with a washout. Neuroendocrine tumours are typically hyperattenuating. Periportal lymphangitic metastasis occurring at the hilum may mimic a cholangiocarcinoma, but they do not show ductal dilatation and involve both sides of the liver [117]. Lymphoepithelioma-like carcinomas are rare malignancies which are associated with Epstein-Barr virus infection. They have an intense lymphocytic infiltrate and may even occur alongside cholangiocarcinomas [118]. Parenchymal hepatic metastasis, that may involve the hilum, is characterized by a central necrosis and hence a hypoattenuating centre. This is rare in CCA. Benign tumours of the bile duct such as neurilemmoma occurring at the hilum may be difficult to differentiate from hilar CCA [119].

## 11. Miscellaneous

Proliferative cholangitis (Cholangitis glandularis proliferans) is a benign intraductal proliferative disorder described by Krukowski et al. in 1983 [120]. It typically involves the extrahepatic biliary tree but may involve the hilum. Proliferative cholangitis has been implicated in hepatolithiasis; whether this is a cause or a consequence is debated. Erdheim-Chester disease is a rare multisystem infiltrative disorder characterized by non-Langhans cell histiocytosis. Biliary hilar infiltration may produce Klatskin-like lesions [121]. Ormond's disease characterized by retroperitoneal fibrosis may present with hilar inflammatory stricture [122]. Heterotopic pancreatic tissue is commonly found in the stomach and duodenum but may occur in the hilar confluence [123] as can heterotopic gastric mucosa [124]. Cholecystohepatic duct with absence of common hepatic duct where the right and left ducts drain into the gall bladder has been mistaken for a malignant hilar stricture on preoperative imaging [125]. Idiopathic nonspecific fibrosis of hilar ducts has also been reported in many series [10, 126, 127].

## 12. Conclusion

In many instances of hilar strictures, resection would probably continue to be the only definitive means of achieving a diagnosis with certainty. This is perhaps justified in comparison to missing a therapeutic window in a potentially operable Klatskin tumour. However, awareness of the varied pathological entities that may mimic a Klatskin tumour and the interpretation of the radiological and pathological data in the clinical context may help identify at least a small proportion of patients who may be deferred the morbidity of surgery. Most such identified patients could be successfully managed by interventional or medical means.

## Figures and Tables

**Table 1:** Incidence of Klatskin-like lesions.

Author (year)	Incidence (%)	Region
Myburgh (1995) [5]	3	South Africa
Verbeek (1992) [7]	13	Netherlands
Gerhards (2001) [8]	15	Netherlands
Knoefel (2003) [9]	18	Germany
Koea (2004) [10]	24	New Zealand
Wetter (1991) [6]	31	California

**Table 2:** Klatskin-like lesions.

(A) Dominant stricture in PSC
(B) Hepatolithiasis and recurrent pyogenic cholangitis
(C) Mirizzi syndrome
(D) Inflammatory-infiltrative
(a) Inflammatory pseudotumour
(b) IgG4 related Cholangiopathy
(c) Eosinophilic cholangiopathy
(d) Follicular cholangiopathy
(e) Xanthogranulomatous cholangitis
(f) Mast cell cholangiopathy
(g) Sarcoidosis
(E) Infective
(a) Cholangiopathy in the immunocompromised
(i) AIDS cholangiopathy
(ii) Primary immunodeficiency
(b) Bacterial
(c) Biliary tuberculosis
(d) Fungal
(e) Parasitic
(F) Vascular
(a) Portal hypertensive biliopathy
(b) Ischaemic cholangiopathy
(G) Toxic
(a) Postchemotherapy
(b) Thorotrast-induced granuloma
(H) Trauma
(a) Biliary
(b) Systemic
(I) Tumours
(a) Malignant
(i) Gall bladder carcinoma
(ii) Hepatocellular carcinoma
(iii) Lymphoepithelioma-like carcinoma
(iv) Neuroendocrine tumours
(v) Granular cell tumour
(vi) Lymphoma
(vii) Leukemia
(viii) Myeloma
(ix) Other metastasis
(b) Benign
(i) Neurilemmoma
(J) Miscellaneous
(a) Proliferative cholangitis
(b) Nonparasitic cysts
(c) Erdheim-Chester disease
(d) Ormond's disease
(e) Heterotopic pancreas/stomach
(f) Cholecystohepatic duct with absent common hepatic duct
(K) Idiopathic

## References

[1] Altemeier WA, Gall EA, Zinninger MM, Hoxworth PI (1957). Sclerosing carcinoma of the major intra hepatic bile ducts. *Archives of Surgery*.

[2] Klatskin G (1965). Adenocarcinoma of the hepatic duct at its bifurcation within the porta hepatis. An unusual tumor with distinctive clinical and pathological features. *The American Journal of Medicine*.

[3] Khan SA, Thomas HC, Davidson BR, Taylor-Robinson SD (2005). Cholangiocarcinoma. *The Lancet*.

[4] Hadjis NS, Collier NA, Blumgart LH (1985). Malignant masquerade at the hilum of the liver. *British Journal of Surgery*.

[5] Myburgh JA (1995). Resection and bypass for malignant obstruction of the bile duct. *World Journal of Surgery*.

[6] Wetter LA, Ring EJ, Pellegrini CA, Way LW (1991). Differential diagnosis of sclerosing cholangiocarcinomas of the common hepatic duct (Klatskin tumors). *American Journal of Surgery*.

[7] Verbeek PC, van Leeuwen DJ, de Wit LT (1992). Benign fibrosing disease at the hepatic confluence mimicking Klatskin tumors. *Surgery*.

[8] Gerhards MF, Vos P, van Gulik TM, Rauws EA, Bosma A, Gouma DJ (2001). Incidence of benign lesions in patients resected for suspicious hilar obstruction. *British Journal of Surgery*.

[9] Knoefel WT, Prenzel KL, Peiper M (2003). Klatskin tumors and Klatskin mimicking lesions of the biliary tree. *European Journal of Surgical Oncology*.

[10] Koea J, Holden A, Chau K, McCall J (2004). Differential diagnosis of stenosing lesions at the hepatic hilus. *World Journal of Surgery*.

[11] Leelawat K, Narong S, Wannaprasert J, Leelawat S (2011). Serum NGAL to clinically distinguish cholangiocarcinoma from benign biliary tract diseases. *International Journal of Hepatology*.

[12] Chen YK, Shah RJ, Pleskow DK (2010). Miami classification (MC) of probe-based confocal laser endomicroscopy (pCLE) findings in the pancreaticobiliary (PB) system for evaluation of indeterminate strictures: interim results from an international multicenter registry. *Gastrointestinal Endoscopy*.

[13] Meining A, Chen YK, Pleskow D (2011). Direct visualization of indeterminate pancreaticobiliary strictures with probe-based confocal laser endomicroscopy: a multicenter experience. *Gastrointestinal Endoscopy*.

[14] Menias CO, Surabhi VR, Prasad SR, Wang HL, Narra VR, Chintapalli KN (2008). Mimics of cholangiocarcinoma: spectrum of disease. *RadioGraphics*.

[15] Lee WJ, Lim H, Jang KM (2001). Radiologic spectrum of cholangiocarcinoma: emphasis on unusual manifestations and differential diagnoses. *RadioGraphics*.

[16] Aljiffry M, Renfrew PD, Walsh MJ, Laryea M, Molinari M (2011). Analytical review of diagnosis and treatment strategies for dominant bile duct strictures in patients with primary sclerosing cholangitis. *International Hepato-Pancreato-Biliary Association*.

[17] Beuers U, Spengler U, Kruis W (1992). Ursodeoxycholic acid for treatment of primary sclerosing cholangitis: a placebo-controlled trial. *Hepatology*.

[18] Nakeeb A, Lipsett PA, Lillemoe KD (1996). Biliary carcinoembryonic antigen levels are a marker for cholangiocarcinoma. *American Journal of Surgery*.

[19] Berstad AE, Aabakken L, Smith HJ, Aasen S, Boberg KM, Schrumpf E (2006). Diagnostic accuracy of magnetic resonance and endoscopic retrograde cholangiography in primary sclerosing cholangitis. *Clinical Gastroenterology and Hepatology*.

[20] Tischendorf JJ, Krüger M, Trautwein C (2006). Cholangioscopic characterization of dominant bile duct stenoses in patients with primary sclerosing cholangitis. *Endoscopy*.

[21] Digby KH (1930). Common-duct stones of liver origin. *British Journal of Surgery*.

[22] Tsui WM, Chan Y, Wong C, Lo Y, Yeung Y, Lee Y (2011). Hepatolithiasis and the syndrome of recurrent pyogenic cholangitis: clinical, radiologic, and pathologic features. *Seminars in Liver Disease*.

[23] Javaid B, Faizallah RM (2001). Intrahepatic stones might mimic cholangiocarcinoma on ERCP. *Gastrointestinal Endoscopy*.

[24] Senda Y, Nishio H, Ebata T (2011). Hepatolithiasis in the hepatic hilum mimicking hilar cholangiocarcinoma: report of a case. *Surgery Today*.

[25] Pack GT, Baker HW (1953). Total right hepatic lobectomy. Report of a case. *Annals of surgery*.

[26] Dehner LP (2004). Inflammatory myofibroblastic tumor: the continued definition of one type of so-called inflammatory pseudotumor. *American Journal of Surgical Pathology*.

[27] Faraj W, Ajouz H, Mukherji D, Kealy G, Shamseddine A, Khalife M (2011). Inflammatory pseudo-tumor of the liver: a rare pathological entity. *World Journal of Surgical Oncology*.

[28] Abdalian R, Heathcote EJ (2006). Sclerosing cholangitis: a focus on secondary causes. *Hepatology*.

[29] Kai K, Matsuyama S, Ohtsuka T, Kitahara K, Mori D, Miyazaki K (2007). Multiple inflammatory pseudotumor of the liver, mimicking cholangiocarcinoma with tumor embolus in the hepatic vein: report of a case. *Surgery Today*.

[30] Yan FH, Zhou KR, Jiang YP, Shi WB (2001). Inflammatory pseudotumor of the liver: 13 cases of MRI findings. *World Journal of Gastroenterology*.

[31] Tublin ME, Moser AJ, Marsh JW, Gamblin TC (2007). Biliary inflammatory pseudotumor: imaging features in seven patients. *American Journal of Roentgenology*.

[32] Zen Y, Nakanuma Y (2012). Ig G4 cholangiopathy. *International Journal of Hepatology*.

[33] Oh HC, Kim MH, Lee KT (2010). Clinical clues to suspicion of IgG4-associated sclerosing cholangitis disguised as primary sclerosing cholangitis or hilar cholangiocarcinoma. *Journal of Gastroenterology and Hepatology*.

[34] Ghazale A, Chari ST, Zhang L (2008). Immunoglobulin G4-associated cholangitis: clinical profile and response to therapy. *Gastroenterology*.

[35] Erkelens GW, Vleggaar FP, Lesterhuis W, van Buuren HR, van der Werf SD (1999). Sclerosing pancreato-cholangitis responsive to steroid therapy. *The Lancet*.

[36] Horiuchi A, Kawa S, Hamano H, Ochi Y, Kiyosawa K (2001). Sclerosing pancreato-cholangitis responsive to corticosteroid therapy: report of 2 case reports and review. *Gastrointestinal Endoscopy*.

[37] Trent V, Khurana KK, Pisharodi LR (1999). Diagnostic accuracy and clinical utility of endoscopic bile duct brushing in the evaluation of biliary strictures. *Archives of Pathology and Laboratory Medicine*.

[38] Kamisawa T, Shimosegawa T, Okazaki K (2009). Standard steroid treatment for autoimmune pancreatitis. *Gut*.

[39] Topazian M, Witzig TE, Smyrk TC (2008). Rituximab therapy for refractory biliary strictures in immunoglobulin G4-associated cholangitis. *Clinical Gastroenterology and Hepatology*.

[40] Albot G, Poilleux F, Oliver C (1949). Les cholécystitis a éosinophiles. *La Presse Médicale*.

[41] Butler TW, Feintuch TA, Caine WP (1985). Eosinophilic cholangitis, lymphadenopathy, and peripheral eosinophilia: a case report. *American Journal of Gastroenterology*.

[42] Nashed C, Sakpal SV, Shusharina V, Chamberlain RS (2010). Eosinophilic cholangitis and cholangiopathy: a sheep in wolves clothing. *HPB Surgery*.

[43] Vauthey JN, Loyer E, Chokshi P, Lahoti S (2003). Case 57: eosinophilic cholangiopathy. *Radiology*.

[44] Aoki T, Kubota K, Oka T, Hasegawa K, Hirai I, Makuuchi M (2003). Follicular cholangitis: another cause of benign biliary stricture. *Hepato-Gastroenterology*.

[45] Lee JY, Lim JH, Lim HK (2005). Follicular cholangitis mimicking hilar cholangiocarcinoma. *Abdominal Imaging*.

[46] Fujita T, Kojima M, Gotohda N (2010). Incidence, clinical presentation and pathological features of benign sclerosing cholangitis of unknown origin masquerading as biliary carcinoma. *Journal of Hepato-Biliary-Pancreatic Sciences*.

[47] Zen Y, Ishikawa A, Ogiso S, Heaton N, Portmann B (2012). Follicular cholangitis and pancreatitis—clinicopathological features and differential diagnosis of an under-recognized entity. *Histopathology*.

[48] Papachristou GI, Demetris AJ, Craig F, Lee KKW, Rabinovitz M (2004). Cholestatic jaundice and bone lesions in an elderly woman. *Nature Clinical Practice Gastroenterology and Hepatology*.

[49] Baron TH, Koehler RE, Rodgers WH, Fallon MB, Ferguson SM (1995). Mast cell cholangiopathy: another cause of sclerosing cholangitis. *Gastroenterology*.

[50] Kwon AH, Matsui Y, Uemura Y (2004). Surgical procedures and histopathologic findings for patients with xanthogranulomatous cholecystitis. *Journal of the American College of Surgeons*.

[51] Kawate S, Ohwada S, Ikota H, Hamada K, Kashiwabara K, Morishita Y (2006). Xanthogranulomatous cholangitis causing obstructive jaundice: a case report. *World Journal of Gastroenterology*.

[52] Kawana T, Suita S, Arima T (1990). Xanthogranulomatous cholecystitis in an infant with obstructive jaundice. *European Journal of Pediatrics*.

[53] Prasil P, Cayer S, Lemay M, Pelletier L, Cloutier R, Leclerc S (1999). Juvenile xanthogranuloma presenting as obstructive jaundice. *Journal of Pediatric Surgery*.

[54] Krishna RP, Kumar A, Singh RK, Sikora S, Saxena R, Kapoor VK (2008). Xanthogranulomatous inflammatory strictures of extrahepatic biliary tract: presentation and surgical management. *Journal of Gastrointestinal Surgery*.

[55] Klatskin G, Yesner R (1950). Hepatic manifestations of sarcoidosis and other granulomatous diseases; a study based on histological examination of tissue obtained by needle biopsy of the liver. *The Yale Journal of Biology and Medicine*.

[56] Ishak KG (1998). Sarcoidosis of the liver and bile ducts. *Mayo Clinic Proceedings*.

[57] Petersen JM (2009). Klatskin-like biliary sarcoidosis: a cholangioscopic diagnosis. *Gastroenterology and Hepatology*.

[58] Onitsuka A, Katagiri Y, Kiyama S (2003). Hilar cholangiocarcinoma associated with sarcoid reaction in the regional lymph nodes. *Journal of Hepato-Biliary-Pancreatic Surgery*.

[59] Karagiannidis A, Karavalaki M, Koulaouzidis A (2006). Hepatic sarcoidosis. *Annals of Hepatology*.

[60] Margulis SJ, Honig CL, Soave R, Govoni AF, Mouradian JA, Jacobson IM (1986). Biliary tract obstruction in the acquired immunodeficiency syndrome. *Annals of Internal Medicine*.

[61] Pol S, Romana CA, Richard S (1993). Microsporidia infection in patients with the human immunodeficiency virus and unexplained cholangitis. *The New England Journal of Medicine*.

[62] Cello JP, Chan MF (1995). Long-term follow-up of endoscopie retrograde cholangiopancreatography sphincterotomy for patients with acquired immune deficiency syndrome papillary stenosis. *American Journal of Medicine*.

[63] Benhamou Y, Caumes E, Gerosa Y (1993). AIDS-related cholangiopathy. Critical analysis of a prospective series of 26 patients. *Digestive Diseases and Sciences*.

[64] Forbes A, Blanshard C, Gazzard B (1993). Natural history of AIDS related sclerosing cholangitis: a study of 20 cases. *Gut*.

[65] Hayward AR, Levy J, Facchetti F (1997). Cholangiopathy and tumors of the pancreas, liver, and biliary tree in boys with X-linked immunodeficiency with hyper-IgM. *Journal of Immunology*.

[66] Rodrigues F, Davies EG, Harrison P (2004). Liver disease in children with primary immunodeficiencies. *Journal of Pediatrics*.

[67] Steinhart AH, Simons M, Stone R, Heathcote J (1990). Multiple hepatic abscesses: cholangiographic changes simulating sclerosing cholangitis and resolution after percutaneous drainage. *American Journal of Gastroenterology*.

[68] Scheppach W, Druge G, Wittenberg G (2001). Sclerosing cholangitis and liver cirrhosis after extrabiliary infections: report on three cases. *Critical Care Medicine*.

[69] Urushihara N, Ariki N, Oyama T (2001). Secondary sclerosing cholangitis and portal hypertension after O157 enterocolitis: extremely rare complications of hemolytic uremic syndrome. *Journal of Pediatric Surgery*.

[70] Alvarez SZ (1998). Hepatobiliary tuberculosis. *Journal of Gastroenterology and Hepatology*.

[71] Chong VH, Telisinghe PU, Yapp SKS, Jalihal A (2009). Biliary strictures secondary to tuberculosis and early ampullary carcinoma. *Singapore Medical Journal*.

[72] Saluja SS, Ray S, Pal S (2007). Hepatobiliary and pancreatic tuberculosis: a two decade experience. *BMC Surgery*.

[73] Özin Y, Parlak E, Kiliç ZM, Temuçin T, Şaşmaz N (2010). Sclerosing cholangitis-like changes in hepatobiliary tuberculosis. *Turkish Journal of Gastroenterology*.

[74] Inal M, Aksungur E, Akgül E, Demirbaş Ö, Oguz M, Erkoçak E (2000). Biliary tuberculosis mimicking cholangiocarcinoma: treatment with metallic biliary endoprothesis. *American Journal of Gastroenterology*.

[75] Albrecht H, Rüsch-Gerdes S, Stellbrink HJ, Greten H, Jäckle S (1997). Disseminated Mycobacterium genavense infection as a cause of pseudo-whipple’s disease and sclerosing cholangitis. *Clinical Infectious Diseases*.

[76] Li KW, Wen TF, Li GD (2010). Hepatic mucormycosis mimicking hilar cholangiocarcinoma: a case report and literature review. *World Journal of Gastroenterology*.

[77] Lin JI, Kabir MA, Tseng HC, Hillman N, Moezzi J, Gopalswamy N (1999). Hepatobiliary dysfunction as the initial manifestation of disseminated cryptococcosis. *Journal of Clinical Gastroenterology*.

[78] Kim JS, Choi BI, Han MC (1994). Cryptococcal cholangiohepatitis with intraductal cryptococcoma. *American Journal of Roentgenology*.

[79] Lefton HB, Farmer RG, Buchwald R, Haselby R (1974). Cryptococcal hepatitis mimicking primary sclerosing cholangitis. A case report. *Gastroenterology*.

[80] Gollan JL, Davidson GP, Anderson K, White TA, Kimber CL (1972). Visceral cryptococcosis without central nervous system or pulmonary involvement: presentation as hepatitis. *Medical Journal of Australia*.

[81] Goenka MK, Mehta S, Yachha SK, Nagi B, Chakraborty A, Malik AK (1995). Hepatic involvement culminating in cirrhosis in a child with disseminated cryptococcosis. *Journal of Clinical Gastroenterology*.

[82] Bucuvalas JC, Bove KE, Kaufman RA (1985). Cholangitis associated with cryptococcus neoformans. *Gastroenterology*.

[83] Hameed H, Sultan F, Khan YI, Hussain M, Azhar R (2009). An unusual cause of obstructive jaundice. *Journal of the Royal College of Physicians of Edinburgh*.

[84] Kim BG, Kang DH, Choi CW (2011). A case of clonorchiasis with focal intrahepatic duct dilatation mimicking an intrahepatic cholangiocarcinoma. *Clinical Endoscopy*.

[85] Dhiman RK, Behera A, Chawla YK, Dilawari JB, Suri S (2007). Portal hypertensive biliopathy. *Gut*.

[86] Condat B, Vilgrain V, Asselah T (2003). Portal cavernoma-associated cholangiopathy: a clinical and MR cholangiography coupled with MR portography imaging study. *Hepatology*.

[87] Chaudhary A, Dhar P, Sachdev A (1998). Bile duct obstruction due to portal biliopathy in extrahepatic portal hypertension: surgical management. *British Journal of Surgery*.

[88] Malkan GH, Bhatia SJ, Bashir K (1999). Cholangiopathy associated with portal hypertension: diagnostic evaluation and clinical implications. *Gastrointestinal Endoscopy*.

[89] Bayraktar Y, Balkanci F, Ozenc A (1995). The “pseudo-cholangiocarcinoma sign” in patients with cavernous transformation of the portal vein and its effect on the serum alkaline phosphatase and bilirubin levels. *American Journal of Gastroenterology*.

[90] Dilawari JB, Chawla YK (1992). Pseudosclerosing cholangitis in extrahepatic portal venous obstruction. *Gut*.

[91] Gorgul A, Kayhan B, Dogan I, Ünal S (1996). Disappearance of the pseudo-cholangiocarcinoma sign after TIPSS. *American Journal of Gastroenterology*.

[92] Bayraktar Y, Ozturk MA, Egesel T, Çekirge S, Balkanci F (2000). Disappearance of “pseudocholangiocarcinoma sign” in a patient with portal hypertension due to complete thrombosis of left portal vein and main portal vein web after web dilatation and transjugular intrahepatic portosystemic shunt. *Journal of Clinical Gastroenterology*.

[93] Khare R, Sikora SS, Srikanth G (2005). Extrahepatic portal venous obstruction and obstructive jaundice: approach to management. *Journal of Gastroenterology and Hepatology*.

[94] Filipponi F, Urbani L, Catalano G (2004). Portal biliopathy treated by liver transplantation. *Transplantation*.

[95] Deltenre P, Valla DC (2008). Ischemic cholangiopathy. *Seminars in Liver Disease*.

[96] Strasberg SM, Helton WS (2011). An analytical review of vasculobiliary injury in laparoscopic and open cholecystectomy. *International Hepato-Pancreato-Biliary Association*.

[97] Ota T, Hirai R, Tsukuda K, Murakam M, Naitou M, Shimizu N (2004). Biliary reconstruction with right hepatic lobectomy due to delayed management of laparoscopic bile duct injuries: a case report. *Acta Medica Okayama*.

[98] Engler S, Elsing C, Flechtenmacher C, Theilmann L, Stremmel W, Stiehl A (2003). Progressive sclerosing cholangitis after septic shock: a new variant of vanishing bile duct disorders. *Gut*.

[99] Gelbmann CM, Rümmele P, Wimmer M (2007). Ischemic-like cholangiopathy with secondary sclerosing cholangitis in critically ill patients. *American Journal of Gastroenterology*.

[100] Saiura A, Umekita N, Inoue S (2001). Benign biliary stricture associated with atherosclerosis. *Hepato-Gastroenterology*.

[101] Ahmed M, Dick M, Mieli-Vergani G, Harrison P, Karani J, Dhawan A (2006). Ischaemic cholangiopathy and sickle cell disease. *European Journal of Pediatrics*.

[102] Barquist ES, Goldstein N, Zinner MJ (1991). Polyarteritis nodosa presenting as a biliary stricture. *Surgery*.

[103] Huong DLT, Valla D, Franco D (1995). Cholangitis associated with paroxysmal nocturnal hemoglobinuria: another instance of ischemic cholangiopathy?. *Gastroenterology*.

[104] Garcia-Tsao G, Korzenik JR, Young L (2000). Liver disease in patients with hereditary hemorrhagic telangiectasia. *The New England Journal of Medicine*.

[105] Viola S, Meyer M, Fabre M (1999). Ischemic necrosis of bile ducts complicating Schonlein-Henoch purpura. *Gastroenterology*.

[106] Chandrasekhara KL, Iyer SK (1984). Obstructive jaundice due to radiation-induced hepatic duct stricture. *American Journal of Medicine*.

[107] Ng SSM, Yu SCH, Lai PBS, Lau WY (2008). Biliary complications associated with selective internal radiation (SIR) therapy for unresectable liver malignancies. *Digestive Diseases and Sciences*.

[108] Makuuchi M, Sukigara M, Mori T (1985). Bile duct necrosis: complication of transcatheter hepatic arterial embolization. *Radiology*.

[109] Park DH, Kim M, Kim TN (2007). Endoscopic treatment for suprapancreatic biliary stricture following blunt abdominal trauma. *American Journal of Gastroenterology*.

[110] Yoon KH, Ha HK, Kim MH (1998). Biliary stricture caused by blunt abdominal trauma: clinical and radiologic features in five patients. *Radiology*.

[111] Osei-Boateng K, Ravendhran N, Haluszka O, Darwin PE (2002). Endoscopic treatment of a post-traumatic biliary stricture mimicking a Klatskin tumor. *Gastrointestinal Endoscopy*.

[112] Schmitt M, Kölbel CB, Müller MK, Verbeke CS, Singer MV (1997). Sclerosing cholangitis after burn injury. *Zeitschrift fur Gastroenterologie*.

[113] Benninger J, Grobholz R, Oeztuerk Y (2005). Sclerosing cholangitis following severe trauma: description of a remarkable disease entity with emphasis on possible pathophysiologic mechanisms. *World Journal of Gastroenterology*.

[114] Hohn D, Melnick J, Stagg R (1985). Biliary sclerosis in patients receiving hepatic arterial infusions of floxuridine. *Journal of Clinical Oncology*.

[115] Botet JF, Watson RC, Kemeny N, Daly JM, Yeh S (1985). Cholangitis complicating intraarterial chemotherapy in liver metastasis. *Radiology*.

[116] Phongkitkarun S, Kobayashi S, Varavithya V, Huang X, Curley SA, Charnsangavej C (2005). Bile duct complications of hepatic arterial infusion chemotherapy evaluated by helical CT. *Clinical Radiology*.

[117] Tada H, Morimoto M, Shima T (1996). Progressive jaundice due to lymphangiosis carcinomatosa of the liver: CT appearance. *Journal of Computer Assisted Tomography*.

[118] Henderson-Jackson E, Nasir NA, Hakam A, Nasir A, Coppola D (2010). Primary mixed lymphoepithelioma-like carcinoma and intra-hepatic cholangiocarcinoma: a case report and review of literature. *International Journal of Clinical and Experimental Pathology*.

[119] Kamani F, Dorudinia A, Goravanchi F, Rahimi F (2007). Extrahepatic bile duct neurilemmoma mimicking Klatskin tumor. *Archives of Iranian Medicine*.

[120] Krukowski ZH, McPhie JL, Farquharson AGH, Matheson NA (1983). Proliferative cholangitis (cholangitis glandularis proliferans). *British Journal of Surgery*.

[121] Gundling F, Nerlich A, Heitland WU, Schepp W (2007). Biliary manifestation of Erdheim-Chester disease mimicking Klatskin’s carcinoma. *American Journal of Gastroenterology*.

[122] Quante M, Appenrodt B, Randerath S, Wolff M, Fischer HP, Sauerbruch T (2009). Atypical Ormond’s disease associated with bile duct stricture mimicking cholangiocarcinoma. *Scandinavian Journal of Gastroenterology*.

[123] Heer C, Pförtner M, Hamberger U, Raute-Kreinsen U, Hanraths M, Bartsch DK (2010). Heterotopic pancreatic tissue in the bifurcation of the bile duct: rare diagnosis mimicking a Klatskin tumour. *Chirurg*.

[124] Kalman PG, Stone RM, Phillips MJ (1981). Heterotopic gastric tissue of the bile duct. *Surgery*.

[125] Dubale N, Anupama NK, Tandon M, Pradeep R, Reddy DN, Rao GV (2011). Anomalous biliary duct mistaken as hilar stricture. A case report. *Journal of Gastroenterology and Hepatology*.

[126] Santoro R, Santoro E, Ettorre GM, Nicolas C, Santoro E (2004). Benign hilar stenosis mimicking Klatskin tumor. *Annales de Chirurgie*.

[127] Uhlmann D, Wiedmann M, Schmidt F (2006). Management and outcome in patients with Klatskin-mimicking lesions of the biliary tree. *Journal of Gastrointestinal Surgery*.

